# Effect of echocardiography on prognosis in patients with type 2 diabetes mellitus

**DOI:** 10.1371/journal.pone.0318153

**Published:** 2025-03-19

**Authors:** Zhi Jiang, Jing-hui Chen, Ling-jie Zhang, You-ping Zheng, Si-hua Qiu

**Affiliations:** Department of Ultrasound, The First Hospital of Longyan, Longyan , Fujian, China; Universitas Indonesia Fakultas Kedokteran, INDONESIA

## Abstract

**Objective:**

There was a lack of studies on the relationship between the presence or absence of echocardiography and the prognosis of type 2 diabetes mellitus (DM) patients. Therefore, we used the Medical Information Mart for Intensive Care (MIMIC)-IV database to explore the relationship between them.

**Method:**

The patient information was obtained from the MIMIC-IV database. Taking age, BMI, sex, race, and marital status as scoring items, Propensity Score Matching was carried out according to the ratio of 1: 1. Generalized linear regression, multivariate logistic regression and hierarchical analysis were used to analyze the correlation between echocardiography and prognosis in type 2 DM patients.

**Results:**

A total of 9140 patients were enrolled in this study. There were differences in body mass index, days of type 2 DM, estimation of glomerular filtration rate, length of stay, survival time, readmission, marital status, family history of type 2 DM, drinking, smoking, metformin, coronary heart disease, heart failure, arrhythmia, hypertension, hyperlipidemia, cardiomyopathy, myocardial infarction, atherosclerosis, epilepsy, and thyroid diseases between patients with echocardiography and those without echocardiography. Echocardiography was independently related to survival time and readmission in type 2 DM patients. Besides, echocardiography was related to the survival time of patients with type 2 DM without complications.

**Conclusions:**

We found for the first time that echocardiography was independently associated with the survival time of type 2 DM patients without complications.

## 1. Introduction

Diabetes mellitus (DM) is a group of metabolic diseases caused by impaired insulin utilization (insulin resistance) or defective secretion (damage to β-cells), with elevated blood glucose levels as the main presenting symptom. It is one of the world’s most challenging public health problems [1, 2]. According to the International Diabetes Federation [3], approximately 537 million adults had DM in 2021. One individual with DM died every five seconds. Additionally, three-quarters of those with DM reside in low- and middle-income countries. It is predicted that about 738 million adults will have DM in 2035. In China, the prevalence of DM has been increasing year by year. The 2015-2017 national survey reported that the prevalence of DM in adults was approximately 11.2%, mainly type 2 DM. The prevalence of DM was significantly higher in men than in women, significantly higher in Han Chinese than in other ethnic groups, higher in urban than in rural areas, an increase in the proportion of undiagnosed DM, and an increase in the prevalence of DM in obese and overweight people [4]. The harm of DM to health is mainly a variety of chronic complications caused by long-term chronic hyperglycemia. The complications of DM mainly include diabetic nephropathy, diabetic eye complications, diabetic foot, diabetic cardiovascular complications, diabetic cerebrovascular diseases, and diabetic neuropathy. More than 70% of people with DM died from cardiovascular and cerebrovascular disease. Diabetic retinopathy caused by DM is the leading cause of blindness in young adults, and kidney damage caused by DM is the main component of advanced end-stage renal disease [5].

Echocardiography is a technique that uses ultrasound waves to produce images of the heart, heart valves, and large blood vessels to help assess the thickness (e.g., hypertrophy or atrophy) and motion of the heart walls and to provide information about ischemia or infarction. Echocardiography can be used to assess the systolic and diastolic function of the left ventricle and to assist in the diagnosis of left ventricular hypertrophy, hypertrophic or restrictive cardiomyopathy, and severe heart failure. Echocardiography is used not only in cardiovascular diseases but also in other diseases.

Liu et al. studied the effect of echocardiography on the long-term prognosis of acute respiratory distress syndrome based on MIMIC data and found that the 90-day mortality rate was significantly lower in the early echocardiography group, and serum lactate levels were significantly lower [6,7]. In patients with multiple organ dysfunction syndrome, it was found that the 28-day mortality rate of patients who had undergone echocardiography examination was significantly reduced [8,9].

At present, most of the research focuses on the relationship between the indicators of echocardiography and type 2 DM, and there is a lack of studies on the relationship between the presence or absence of echocardiography and the clinical prognosis of type 2 DM patients. In addition, type 2 DM patients generally experienced unfavorable complications. Whether their correlation is consistent among patients with different complications is still unknown. Therefore, we used the MIMIC-IV database to explore the relationship between them.

## 2. Methods

### 2.1. Data source

The patient information was obtained from the MIMIC-IV database (https://mimic.mit.edu/), which is currently the largest publicly accessible clinical database in the field of emergency and critical care.

### 2.2. Inclusion and exclusion criteria

The inclusion criteria for this study were patients aged ≥  18 years, hospitalized time ≥  2 days, and diagnosed with type 2 DM.

The exclusion criteria for this study was the patient was diagnosed with gestational DM.

### 2.3. Indicators

The main variable was whether to do echocardiography.

The prognosis indicators were survival time, length of stay (LOS), ICU readmission (no, yes), readmission (no, yes) and death (no, yes). Death in this article was the outcome of follow-up.

Other indicators mainly included age, body mass index (BMI), days of type 2 DM, estimation of glomerular filtration rate (eGFR), systolic blood pressure (SBP), diastolic blood pressure (DBP), race (white, other), sex (male, female), marital status (single, married, divorced, widowed), family history of type 2 DM (no, yes), drinking (no, yes), smoking (no, yes), metformin (no, yes), coronary heart disease (no, yes), heart failure (no, yes), arrhythmia (no, yes), hypertension (no, yes), hyperlipidemia (no, yes), cardiomyopathy (no, yes), myocardial infarction (no, yes), atherosclerosis (no, yes), epilepsy (no, yes), thyroid diseases (no, yes).

### 2.4. Statistical analysis

The R language was employed for data cleansing and analysis. Taking age, BMI, sex, race, and marital status as scoring items, Propensity Score Matching (PSM) was carried out according to the ratio of 1: 1. Non-normally distributed quantitative data were statistically described using the median (interquartile range) and the Mann-Whitney U test was used to compare between the two groups. Qualitative data were statistically described using the component ratios and the chi-square test was used to compare between the two groups. Generalized linear regression or multivariate logistic regression was used to analyze the correlation between the echocardiography and prognosis in type 2 DM patients after adjusting confounding variables. Generalized linear regression was also used to analyze the correlation between the echocardiography and prognosis in type 2 DM patients with complications or without complications in different models (crude model, adjusted model). Hierarchical analysis was employed to analyze the correlation between the echocardiography and prognosis in type 2 DM patients with different complications in different models (crude model, adjusted model). P <  0.05 was considered to be statistically significant.

## 3. Results

### 3.1. Patient baseline information

After PSM, A total of 9140 patients were enrolled in this study. Table 1 showed that there were differences in BMI, days of type 2 DM, eGFR, LOS, survival time, readmission, marital status, family history of type 2 DM, drinking, smoking, metformin, coronary heart disease, heart failure, arrhythmia, hypertension, hyperlipidemia, cardiomyopathy, myocardial infarction, atherosclerosis, epilepsy, and thyroid diseases between patients with echocardiography and those without echocardiography (all P <  0.05).

**Table 1.** The baseline data of patients.

Variable		Without echocardiography patient	With echocardiography patient	P
Age		66.000 [57.000,76.000]	66.000 [57.000,75.000]	0.079
BMI		30.400 [26.000,35.700]	30.900 [26.600,36.200]	<0.001
Days of type 2 DM		10.903 [5.018,95.885]	17.550 [5.625,248.797]	<0.001
eGFR		1.005 [0.806,1.049]	0.993 [0.801,1.043]	<0.001
SBP		110.000 [102.000,122.000]	110.000 [102.000,122.000]	0.819
DBP		64.000 [58.000,72.000]	64.000 [58.000,72.000]	0.716
LOS		5.692 [3.778,9.474]	5.845 [3.953,9.523]	0.002
Survival time		157.000 [46.092,342.353]	222.560 [58.056,569.157]	<0.001
ICU readmission	No	4405 (96.389)	4417 (96.652)	0.493
	Yes	165 (3.611)	153 (3.348)	
Readmission	No	2517 (55.077)	1892 (41.400)	<0.001
	Yes	2053 (44.923)	2678 (58.600)	
Death	No	850 (91.202)	1375 (90.342)	0.477
	Yes	82 (8.798)	147 (9.658)	
Race	White	2816 (62.260)	2690 (60.287)	0.055
	Other	1707 (37.740)	1772 (39.713)	
Sex	Male	2460 (53.829)	2377 (52.013)	0.082
	Female	2110 (46.171)	2193 (47.987)	
Marital status	Single	1231 (27.085)	1369 (30.402)	<0.001
	Married	2229 (49.043)	1965 (43.638)	
	Divorced	414 (9.109)	418 (9.283)	
	Widowed	671 (14.763)	751 (16.678)	
Family history of type 2 DM	No	4224 (92.429)	4291 (93.895)	0.005
	Yes	346 (7.571)	279 (6.105)	
Drinking	No	4260 (93.217)	4175 (91.357)	<0.001
	Yes	310 (6.783)	395 (8.643)	
Smoking	No	4458 (97.549)	4416 (96.630)	0.009
	Yes	112 (2.451)	154 (3.370)	
Metformin	No	3677 (80.460)	4021 (87.987)	<0.001
	Yes	893 (19.540)	549 (12.013)	
Coronary heart disease	No	2843 (62.210)	2499 (54.683)	<0.001
	Yes	1727 (37.790)	2071 (45.317)	
Heart failure	No	3145(68.818)	2331 (51.007)	<0.001
	Yes	1425(31.182)	2239 (48.993)	
Arrhythmia	No	4378 (95.799)	4334 (94.836)	0.029
	Yes	192 (4.201)	236 (5.164)	
Hypertension	No	1499 (32.801)	861 (18.840)	<0.001
	Yes	3071 (67.199)	3709 (81.160)	
Hyperlipidemia	No	1446 (31.641)	1000 (21.882)	<0.001
	Yes	3124 (68.359)	3570 (78.118)	
Cardiomyopathy	No	4164 (91.116)	3951 (86.455)	<0.001
	Yes	406 (8.884)	619 (13.545)	
Myocardial infarction	No	3539 (77.440)	3141 (68.731)	<0.001
	Yes	1031 (22.560)	1429 (31.269)	
Atherosclerosis	No	3925 (85.886)	2964 (64.858)	<0.001
	Yes	645 (14.114)	1606 (35.142)	
Epilepsy	No	4440 (97.155)	4362 (95.449)	<0.001
	Yes	130 (2.845)	208 (4.551)	
Cerebrovascular disease	No	4548 (99.519)	4538 (99.300)	0.172
	Yes	22 (0.481)	32 (0.700)	
Thyroid diseases	No	3815 (83.479)	3677 (80.460)	<0.001
	Yes	755 (16.521)	893 (19.540)	

LOS: length of stay, BMI: body mass index, eGFR: estimation of glomerular filtration rate, SBP: systolic blood pressure, DBP: diastolic blood pressure,

### 3.2. Relationship between echocardiography and prognosis in type 2 DM patients

The results of the analysis of the baseline data showed that LOS, survival time, and readmission of the prognosis indicators differed between patients who underwent echocardiography and those who did not, so we further analyzed the correlation between them in type 2 DM patients by generalized linear regression or multivariate logistic regression after adjusting for significant baseline features. As can be seen from Table 2, echocardiography was not independently associated with LOS in type 2 DM patients. The results in Table 3 and Table 4 showed that echocardiography was independently related to survival time and readmission in type 2 DM patients.

**Table 2:** The relationship between echocardiography and LOS in type 2 DM population.

Predictor	β (95%CI)	P
(Intercept)	10.198 (7.786,12.61)	<0.001
BMI	0.001 (-0.002,0.003)	0.501
Days of type 2 DM	-0.001 (-0.002,0.000)	0.247
eGFR	-1.577 (-3.968,0.815)	0.196
Echocardiography	0.066 (-0.564,0.695)	0.838
Family history of type 2 DM	-0.832 (-1.901,0.236)	0.127
Metformin	-0.006 (-0.723,0.710)	0.986
Coronary heart disease	0.699 (-0.987,2.384)	0.417
Heart failure	-0.031 (-0.794,0.732)	0.936
Arrhythmia	1.204 (-0.299,2.707)	0.116
Hypertension	0.267 (-0.33,0.864)	0.381
Hyperlipidemia	-0.234 (-0.829,0.361)	0.440
Cardiomyopathy	-0.064 (-1.12,0.992)	0.906
Myocardial infarction	0.258 (-0.549,1.065)	0.532
Atherosclerosis	0.773 (-0.224,1.769)	0.129
Epilepsy	-0.250 (-1.787,1.287)	0.750
Thyroid diseases	-0.461 (-1.213,0.292)	0.230
Married	0.140 (-0.518,0.799)	0.677
Divorced	-0.406 (-1.455,0.642)	0.447
Widowed	-0.427 (-1.355,0.501)	0.367
Drinking	-0.285 (-1.347,0.777)	0.599
Smoking	-0.372 (-2.041,1.297)	0.662

LOS: length of stay, BMI: body mass index, eGFR: estimation of glomerular filtration rate,

**Table 3:** The relationship between echocardiography and survival time in type 2 DM population.

Predictor	β (95%CI)	P
(Intercept)	81.942 (-9.433,173.316)	0.079
BMI	0.036 (-0.060,0.132)	0.465
Days of type 2 DM	0.949 (0.909,0.988)	<0.001
eGFR	20.388 (-70.819,111.596)	0.661
Echocardiography	31.216 (9.893,52.539)	0.004
Family history of type 2 DM	-6.671 (-50.118,36.775)	0.764
Metformin	27.118 (-12.001,66.237)	0.175
Coronary heart disease	48.913 (-4.346,102.173)	0.072
Heart failure	10.570 (-15.047,36.187)	0.419
Arrhythmia	-44.660 (-121.334,32.014)	0.254
Hypertension	-1.530 (-22.940,19.88)	0.889
Hyperlipidemia	7.885 (-13.905,29.675)	0.478
Cardiomyopathy	-15.875 (-49.043,17.292)	0.348
Myocardial infarction	-19.742 (-46.001,6.517)	0.141
Atherosclerosis	37.118 (4.563,69.673)	0.026
Epilepsy	-10.450 (-59.054,38.153)	0.674
Thyroid diseases	23.676 (-2.783,50.135)	0.08
Married	-23.829 (-49.293,1.636)	0.067
Divorced	7.974 (-31.725,47.673)	0.694
Widowed	4.391 (-26.636,35.418)	0.782
Drinking	-16.831 (-52.613,18.952)	0.357
Smoking	-7.947 (-69.091,53.196)	0.799

BMI: body mass index, eGFR: estimation of glomerular filtration rate,

**Table 4:** The relationship between echocardiography and readmission in type 2 DM population.

Predictor	OR (95%CI)	P
(Intercept)	0.300 (0.165,0.543)	<0.001
BMI	1.000 (0.999,1.001)	0.894
Days of type 2 DM	1.005 (1.004,1.005)	<0.001
eGFR	1.604 (0.889,2.895)	0.117
Echocardiography	0.794 (0.677,0.930)	0.004
Family history of type 2 DM	0.751 (0.570,0.984)	0.039
Metformin	0.816 (0.681,0.975)	0.026
Coronary heart disease	2.773 (1.737,4.484)	<0.001
Heart failure	1.615 (1.343,1.944)	<0.001
Arrhythmia	0.586 (0.389,0.872)	0.009
Hypertension	0.774 (0.670,0.896)	0.001
Hyperlipidemia	1.240 (1.073,1.435)	0.004
Cardiomyopathy	1.050 (0.804,1.372)	0.718
Myocardial infarction	1.164 (0.954,1.419)	0.135
Atherosclerosis	1.661 (1.293,2.136)	<0.001
Epilepsy	0.957 (0.660,1.376)	0.815
Thyroid diseases	1.062 (0.882,1.276)	0.526
Married	0.810 (0.689,0.952)	0.011
Divorced	0.833 (0.643,1.077)	0.166
Widowed	0.977 (0.779,1.225)	0.842
Drinking	1.136 (0.874,1.473)	0.337
Smoking	1.378 (0.917,2.063)	0.120

BMI: body mass index, eGFR: estimation of glomerular filtration rate,

### 3.3. Relationship between the echocardiography and prognosis in type 2 DM patients with complications

The main harm of DM comes from complications, which is the main cause of disability and death of DM patients. Therefore, we further explored the relationship between echocardiography and prognoses in type 2 DM patients with complications. As can be seen from Table 5, in the crude model, echocardiography was not only related to the survival time of patients with type 2 DM without complications but also related to the survival time of type 2 DM patients with complications. However, in the adjusted model, echocardiography was only related to the survival time of patients with type 2 DM without complications. The same results were found in Table 6 in the crude model, after adjusting all confounding factors, there was no correlation between echocardiography and readmission of type 2 DM patients without complications and type 2 DM patients with complications.

**Table 5:** Relationship between the echocardiography and survival time in type 2 DM patients with or without complications.

	Crude		Adjusted	
	β (95%CI)	P	β (95%CI)	P
Total	126.212 [96.292,156.132]	<0.001	31.171 [9.845,52.497]	0.004
Complications				
Without	151.580 [104.015,199.145]	<0.001	53.846 [21.081,86.612]	0.001
With	108.573 [70.090,147.057]	<0.001	7.725 [-21.329,36.779]	0.602

Adjusting BMI, days of type 2 DM, eGFR, family history of type 2 DM, metformin, coronary heart disease, heart failure, arrhythmia, hypertension, hyperlipidemia, cardiomyopathy, myocardial infarction, atherosclerosis, epilepsy, thyroid diseases, marital status, drinking, smoking

**Table 6:** Relationship between the echocardiography and readmission in type 2 DM patients with or without complications.

	Crude		Adjusted	
	OR (95%CI)	P	OR (95%CI)	P
Total	1.735 [1.597,1.885]	<0.001	0.814 [0.696,0.953]	0.011
Complications				
Without	2.104 [1.856,2.384]	<0.001	1.017 [0.809,1.279]	0.884
With	1.695 [1.501,1.915]	<0.001	0.868 [0.685,1.100]	0.241

Adjusting BMI, days of type 2 DM, eGFR, family history of type 2 DM, metformin, coronary heart disease, heart failure, arrhythmia, hypertension, hyperlipidemia, cardiomyopathy, myocardial infarction, atherosclerosis, epilepsy, thyroid diseases, marital status, drinking, smoking

We included 5 types of complications of type 2 DM with the number of patients ≥  30 for further study. We further explored the precise independent role of echocardiography on the survival time among type 2 DM patients with different complications after adjusting. Table 7 showed that echocardiography was not correlated with the survival time in subgroup type 2 DM patients with complications.

**Table 7:** Relationship between the echocardiography and survival time in type 2 DM patients with 5 complications.

	Crude		Adjusted	
Complications	β (95%CI)	P	β (95%CI)	P
Hypoglycemia without coma	54.477 [-118.846,227.800]	0.538	5.530 [-114.837,125.896]	0.928
Diabetic peripheral angiopathy without gangrene	7.329 [-90.890,105.549]	0.884	72.201 [-103.420,247.821]	0.420
Unspecified diabetic neuropathy	148.570 [-12.781,309.922]	0.071	-17.709 [-107.858,72.441]	0.700
Diabetic nephropathy	4.490 [-192.629,201.608]	0.964	-109.570 [-342.663,123.523]	0.357
Diabetic chronic kidney disease	83.373 [32.346,134.400]	0.001	1.766 [-40.644,44.176]	0.935

Adjusting BMI, days of type 2 DM, eGFR, family history of type 2 DM, metformin, coronary heart disease, heart failure, arrhythmia, hypertension, hyperlipidemia, cardiomyopathy, myocardial infarction, atherosclerosis, epilepsy, thyroid diseases, marital status, drinking, smoking

## 4. Discussion

We made a retrospective analysis of type 2 DM patients based on the MIMIC-IV database and found for the first time that echocardiography was independently associated with survival time and readmission in type 2 DM patients. In addition, we also found that there was a positive correlation between echocardiography and the survival time of type 2 DM patients without complications.

Compared with the without echocardiography group, the proportion of drinking, smoking, coronary heart disease, heart failure, arrhythmia, hyperlipidemia, cardiomyopathy, myocardial infarction, atherosclerosis, epilepsy, and thyroid diseases in the echocardiography group was higher in this study. In similar studies, it was also found that patients in the echocardiography group had a larger proportion of chronic diseases [10]. We found that BMI was higher, days of type 2 DM were longer and eGFR was lower in the echocardiography group. Overweight patients have a higher incidence of cardiovascular diseases [11]. A longer duration of DM was found to be associated with an increased risk of heart failure [12]. Higher eGFR was linked to a reduction in cardiovascular disease across all age groups [13]. We suspected that this situation in the echocardiography group may be related to the doctor’s suggestion to the patient for echocardiography according to the patient’s situation and disease.

This study found that echocardiography was independently related to survival time and readmission in type 2 DM patients which suggested that echocardiography affected the prognosis of patients. Hu et al. conducted research based on the MIMIC-III database and found that echocardiography can reduce the 28-day mortality of patients with organ failure [9]. Dong et al. included 1346 patients with acute aspiration distress syndrome and found that the 28-day mortality rate of patients with echocardiography was significantly lower than that of patients without echocardiography [14]. It is found that echocardiography can improve the 28-day mortality of patients with acute kidney injury, septic shock, and elderly critically ill patients [15–17]. European guidelines suggest that echocardiography should also be regarded as a diagnostic test for patients with type 2 DM regardless of whether they have cardiovascular disease [18]. It was found that 1/4 of patients with type 2 DM had abnormal electrocardiogram in the outpatient department of the secondary hospital [19]. Therefore, it is very necessary to carry out echocardiography for type 2 DM people. This study found that there is a positive correlation between echocardiography and the survival time of DM patients, suggesting that the survival time of type 2 DM patients will be increased by echocardiography. In addition, echocardiography was a protective factor for the readmission of patients with type 2 DM in this study. Our results proved that type 2 DM patients should perform echocardiography. At present, the research on type 2 DM and echocardiography mainly focuses on predicting the adverse events of type 2 DM with the related indexes of echocardiography. Jørgensen et al. performed echocardiography on 933 patients with type 2 DM and found that the average E/E’ was the strongest predictor of future cardiovascular events in DM men, while Global longitudinal strain was the strongest predictor of future cardiovascular events in women [20]. Another study found a correlation between coronary flow reserve (CFR) measured by echocardiography and prognosis in asymptomatic patients with type 2 DM without significant coronary artery disease, with asymptomatic patients with type 2 DM with a CFR < 2.5 having a worse prognosis [21, 22]. Another study based on the medical center database found that E/A ratio, E/E’ ratio, and tricuspid regurgitation velocity were related to the risk factors of death in type 2 DM [14,23].

In this study, we found there is a positive correlation between echocardiography and the survival time of DM patients. We suspected that this may be related to the fact that after echocardiography, the patient was found to have some cardiovascular diseases and then changed their habits, thus prolonging the survival time. Studies have found that drinking and smoking impair cardiac systolic and diastolic function [15–17]. After echocardiography was performed and a problem with the heart was detected, the patient may undergo smoking and alcohol cessation, to achieve a better quality of life. In addition, doctors would treat patients with cardiovascular diseases according to the results of echocardiography, which was conducive to controlling the occurrence and development of diseases and thus prolonging the survival time [24]. Our study found that the readmission rate of patients with echocardiography was higher than that of patients without echocardiography, which may be related to their diseases. From the baseline data, we can easily find that the prevalence rate of basic diseases in patients with echocardiography was higher. We also found that echocardiography was positively correlated with the survival time of type 2 DM patients without complications. This suggested that in the daily check-up, this part of the population can be examined by echocardiography in a targeted manner. We did not find a relationship between echocardiography and complications of type 2 DM, which may be related to the small number of people with complications in this study.

This study has some limitations. First, because of the database, we can only know whether the patient has performed echocardiography, and we can’t further study it according to the indicators of echocardiography. Secondly, the objective of this study was to examine the association between whether echocardiography was performed and survival time in the type 2 DM population. Therefore, this study did not compare with healthy people. Finally, due to the sample size, we can only study the correlation between the survival time and whether the patients with complications of 5 types of type 2 DM underwent echocardiography.

## 5. Conclusions

We found for the first time that echocardiography was independently associated with the survival time of type 2 DM patients without complications.

## Supporting information

S1 Data Raw data after matching. (XLSX)

S2 Data Raw data before matching. (XLS)
